# Supplementary material for: Assessment of Cytokine-Induced Neutrophil Chemoattractants as Biomarkers for Prediction of Pulmonary Toxicity of Nanomaterials
Source: Nanomaterials (Basel). 2020 Aug 9;10(8):1563. doi: 10.3390/nano10081563 (PMC7466583; doi:10.3390/nano10081563)
Supplement: Supplementary file 1 [file nanomaterials-10-01563-s001.pdf]

nanomaterials

MDPI

Article

Assessment of Cytokine-Induced Neutrophil Chemoattractants as Biomarkers for Prediction of Pulmonary Toxicity of Nanomaterials

Taisuke Tomonaga [1,*], Hiroto Izumi [1], Takako Oyabu [1], Byeong-Woo Lee [1], Masaru Kubo [2], Manabu Shimada [2], Shingo Noguchi [3], Chinatsu Nishida [3], Kazuhiro Yatera [3] and Yasuo Morimoto [1]

1 Institute of Industrial Ecological Sciences, University of Occupational and Environmental Health, 1-1 Iseigaoka, Yahata-nishi-ku, Kitakyushu, Fukuoka 807-8555, Japan; h-izumi@med.uoeh-u.ac.jp (H.I.); toyabu@med.uoeh-u.ac.jp (T.O.); leebw401@med.uoeh-u.ac.jp (B.-W.L.); yasuom@med.uoeh-u.ac.jp (Y.M.)

2 Department of Chemical Engineering, Graduate School of Engineering, Hiroshima University, 4-1 Kagamiyama 1-chome, Higashi-Hiroshima-shi, Hiroshima 739-8527, Japan; mkubo@hiroshima-u.ac.jp (M.K.); smd@hiroshima-u.ac.jp (M.S.)

3 Department of Respiratory Medicine, University of Occupational and Environmental Health, 1-1 Iseigaoka, Yahata-nishi-ku, Kitakyushu, Fukuoka, 807-8555, Japan; sn0920@med.uoeh-u.ac.jp (S.N.); c-nishi@med.uoeh-u.ac.jp (C.N.); yatera@med.uoeh-u.ac.jp (K.Y.)

* Correspondence: t-tomonaga@med.uoeh-u.ac.jp; Tel.: +81-93-691-7466

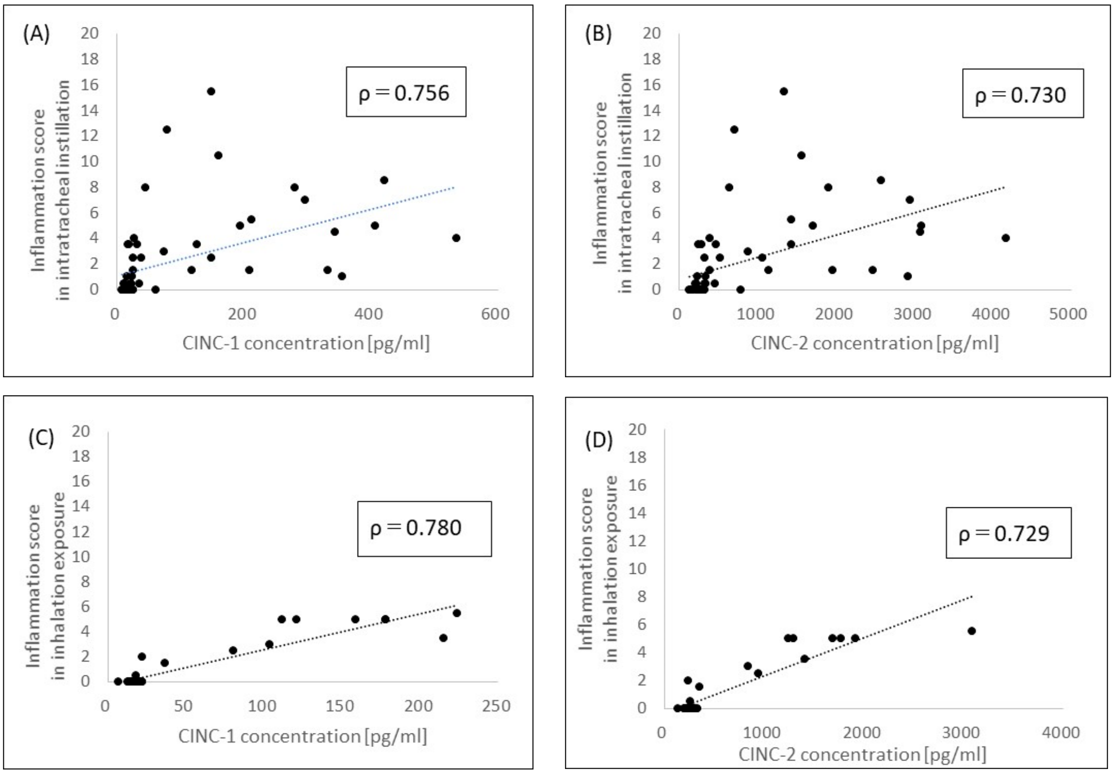

Figure S1. Relationship between CINCs and inflammation scores: (A) CINC-1 in intratracheal instillation, (B) CINC-2 in intratracheal instillation, (C) CINC-1 in inhalation exposure and (D) CINC-2 in inhalation exposure versus inflammation scores in the histopathological findings of the lung at each observation time. CINC-1 and CINC-2 had positive correlation with inflammation scores of histopathological findings after both of intratracheal instillation and inhalation exposure. Values of ρ are Spearman's rank correlation coefficient for all the data.

Table S1. Characterization of inhaled chemicals including NMs.

Samples	Toxicity	Exposure route	Characterization	Animal (Rat)	Negative control	Dose/ Concentration	Lung inflammation	Reference
NiO	High	IT/IH	Size 19 nm, BET 57 m^2/g<br>Secondary particle diameter (DLS) 59.7 nm	Male Fischer344	Distilled water/ Clean air	0.2 mg/rat, 1.0 mg /rat<br>0.32 ± 0.07 mg/m^3, 1.65 ± 0.20 mg/m^3	+/±	[19]
CeO_2	High	IT/IH	Size 7.8 nm, BET 101 m^2/g<br>Secondary particle diameter (DLS) 10.0 nm	Male Fischer344	Distilled water/ Clean air	0.2 mg/rat, 1.0 mg/rat<br>2.09 ± 0.29 mg/m^3, 10.2 ± 1.38 mg/m^3	+/+	[21]
TiO_2(P90)	Low	IT	Size 14 nm, BET 104 m^2/g<br>Secondary particle diameter (DLS) 22.7 nm	Male Wistar	Distilled water	0.2 mg/rat, 1.0 mg/rat	±	[20]
TiO_2(Rutile)	Low	IT/IH	Size 12 nm × 55 nm, BET 111 m^2/g<br>Secondary particle diameter (DLS) 44.9 nm	Male Fischer344	Distilled water/ Clean air	0.2 mg/rat, 1.0 mg/rat<br>0.50 ± 0.26 mg/m^3, 1.84 ± 0.74 mg/m^3	±/−	[19]
ZnO	Low	IT/IH	Size 35 nm, BET 31 m^2/g<br>Secondary particle diameter (DLS) 33 nm	Male Fischer344	Distilled water/ Clean air	0.2 mg/rat, 1.0 mg/rat<br>2.11 ± 0.45 mg/m^3, 10.4 ± 1.39 mg/m^3	±/±	[22]
SiO_2	High	IT	Primary particle size 1.6 μm,	Male Fischer344	Distilled water	1.0 mg/rat	+	[23]

IT: Intratracheal instillation, IH: Inhalation exposure.
Lung inflammation +: persistent inflammation, ±: transient inflammation, −: no inflammation.

Table S2. Summaries of the neutrophil counts in BALF after intratracheal instillation and inhalation exposure of NMs.

**Neutrophil counts in BALF (×1000 cells/mL±SD)**							
	**Dose/Concentration**		**3 days**	**1 week**	**1 month**	**3 months**	**6 months**
**Intratracheal instillation**							
Negative control			2.88 ± 1.58	0.22 ± 0.49	0.20 ± 0.45	0.08 ± 0.17	0.69 ± 1.53
NiO	Low	0.2 mg/rat	21.1 ± 8.74*	78.93 ± 18.40**	52.38 ± 12.34**	13.03 ± 12.13	1.27 ± 1.97
NiO	High	1.0 mg/rat	153.5 ± 44.6**	158.51 ± 56.43**	161.69 ± 67.27*	279.80 ± 125.57*	59.80 ± 15.28**
Negative control			0.22 ± 0.25	0.20 ± 0.22	0.26 ± 0.47	0.25 ± 0.35	1.13 ± 1.19
TiO_2 (P90)	Low	0.2 mg/rat	0.80 ± 0.55	0.31 ± 0.21	0.65 ± 0.46	0.53 ± 0.50	2.36 ± 3.82
TiO_2 (P90)	High	1.0 mg/rat	54.50 ± 31.86*	20.35 ± 13.61	2.89 ± 1.97	0.32 ± 0.35	0.49 ± 0.42
Negative control			1.73 ± 10.6	0.80 ± 0.73	4.74 ± 2.08	0.57 ± 0.80	0.28 ± 0.39
TiO_2 (Rutile)	Low	0.2mg/rat	11.95 ± 4.94	1.61 ± 1.29	1.81 ± 1.13	11.07 ± 22.57	1.62 ± 2.88
TiO_2 (Rutile)	High	1.0mg/rat	174.7 ± 121.8*	110.33 ± 39.14**	5.27 ± 0.98	105.21 ± 229.82	0.35 ± 0.51
Negative control			6.50 ± 5.17	1.76 ± 0.96	0.90 ± 1.32	0.45 ± 0.49	1.37 ± 2.26

CeO_2	Low	0.2mg/rat	111.66 ± 48.09*	119.52 ± 79.79*	27.17 ± 15.26*	10.93 ± 3.25**	1.91 ± 1.39
CeO_2	High	1.0mg/rat	170.52 ± 35.04**	234.21 ± 55.62**	74.65 ± 6.72**	56.73 ± 15.54**	11.07 ± 1.88**
Negative control			2.35 ± 0.41	0.47 ± 0.36	1.08 ± 0.32	1.12 ± 0.81	3.55 ± 1.92
ZnO	Low	0.2mg/rat	191.38 ± 42.19**	7.032 ± 1.61**	1.02 ± 0.94	3.21 ± 2.23	3.10 ± 1.32
ZnO	High	1.0mg/rat	395.82 ± 78.47**	11.44 ± 8.65	0.98 ± 1.28	1.73 ± 1.66	4.20 ± 3.36
**Inhalation exposure**							
Negative control			1.09 ± 2.43		14.23 ± 22.90	0.35 ± 0.77	
NiO	Low	0.32 ± 0.07 mg/m^3	1.39 ± 3.12		3.67 ± 2.84	1.30 ± 1.44	
NiO	High	1.65 ± 0.20 mg/m^3	84.10 ± 54.54		21.97 ± 12.84	1.29 ± 1.43	
Negative control			0 ± 0		0.09 ± 0.19	0 ± 0	
TiO_2 (Rutile)	Low	0.50 ± 0.26 mg/m^3	0.15 ± 0.20		0 ± 0	0.48 ± 1.07	
TiO_2 (Rutile)	High	1.84 ± 0.74 mg/m^3	0.12 ± 0.27		0.18 ± 0.19	0 ± 0	
Negative control			0.55 ± 0.54		1.65 ± 0.64	1.94 ± 1.10	
CeO_2	Low	2.09 ± 0.29 mg/m^3	38.22 ± 6.21**		19.70 ± 7.38*	11.13 ± 2.77**	
CeO_2	High	10.2 ± 1.38 mg/m^3	96.74 ± 40.54*		114.92 ± 72.26	49.18 ± 16.35**	
Negative control			2.24 ± 1.95		0.56 ± 0.62	1.77 ± 0.66	
ZnO	Low	2.11 ± 0.45 mg/m^3	1.91 ± 0.63		0 ± 0	2.50 ± 0.49	
ZnO	High	10.4 ± 1.39 mg/m^3	126.06 ± 45.21**		0.99 ± 2.17	2.24 ± 0.81	

Significant differences compared with each control (*$p<0.05$, **$p<0.01$).

© 2020 by the authors. Submitted for possible open access publication under the terms and conditions of the Creative Commons Attribution (CC BY) license (http://creativecommons.org/licenses/by/4.0/).
